# Assessing the efficacy of the ‘Bicho De 7 Cabeças’ B-learning school-based program in enhancing mental health literacy and reducing stigma

**DOI:** 10.1186/s40359-024-01591-2

**Published:** 2024-02-23

**Authors:** Gislene Meilsmeidth, Maria João Trigueiro, Vítor Simões-Silva, Raquel Simões de Almeida, Paula Portugal, Paulo Veloso Gomes, Sara de Sousa, Filipa Campos, Pedro Monteiro, Ana Paula Soutelo, António Marques

**Affiliations:** LabRP– CIR, ESS, Polytechnic University of Porto, Rua Dr. António Bernardino de Almeida, 400 4200 - 072 Porto, Portugal

**Keywords:** Stigma, Mental health literacy, B-learning, School-based program, Youth

## Abstract

The prevalence of mental disorders in adolescents has a considerable impact on daily life, restricting tasks and diminishing overall quality of life while potentially leading to stigmatization. This study aims to measure the impact of a mental health literacy intervention program, called “Bicho de 7 Cabeças” project, in b-learning format, on the increase of knowledge and the decrease of stigma in young people from Póvoa de Varzim, in Portugal. A quasi-experimental study was conducted, from November 2022 to May 2023, involving an experimental group (“Bicho de 7 Cabeças” protocol) and an active control group (informational brochures), utilizing a pre-test/post-test design. Mental Health Literacy Measure-MHLM, Mental Health Promoting Knowledge Scale-MHPK-10, Mental Illness Knowledge Schedule-MAKS, Reported and Intended Behaviour Scale-RIBS, and Community Attitudes toward People with Mental Illness-CAMI were used. A total of 504 young students from the 9th grade enroll in this study, with a mean age around 14 years old. There is a significant difference between stigma (*p* <.001) and knowledge (*p* <.001) scores at baseline and follow-up. The results of this study shows that interventions aimed at young people for the promotion of mental health literacy and stigma reduction are needed and more initiatives should be implemented in schools to address these problems.

**Supplementary Information:**

The online version contains supplementary material available at 10.1186/s40359-024-01591-2.

## Introduction

Globally, there is a prevalence of about 10 to 20% of children and adolescents who suffer from a mental disorder [1], with the most prevalent being attention deficit hyperactivity disorder (ADHD) 3.4%, conduct disorder 5.7%, substance use disorders 13.6%, depression 2.6% and anxiety 6.5% [2, 3]. In Portugal, the 2021/2022 Health Behaviourin School– Aged Children study showed a worrying deterioration in the mental health and general well-being of Portuguese adolescents, particular with higher rates of sadness, self-harm, and psychological and physical symptoms are indicators of this deterioration; a total of 5809 young people, with a mean age of 14.09 years, participated in the study [4]. It is therefore essential to promote social and emotional learning and psychological well-being, and to ensure access to mental health care, which are essential for health and well-being during adolescence and adulthood [5]. However, there is a large discrepancy between the need and demand for mental health services and their availability by the young population. There are multiple factors, both personal and environmental, that prevent people from seeking mental health care, from limited access to services, low economic resources, stigma, and low mental health literacy (MHL). Several studies show that low MHL in the general population is one of the factors that makes difficult to seek help and access treatment [6–10]. Some authors add that the low level of MHL also leads to the development of unfounded beliefs about different mental disorders, which is more evident in the young population [11].

Mental health literacy emerged in Australia from the concept of health literacy. The authors defined the concept of MHL as the knowledge and beliefs about mental disorders that leads to the adoption of behaviors that promote mental health and the prevention or proper management of existing disorders [12]. MHL is, therefore, a key construct for the promotion of mental health, prompting the individuals for action decision-making about their mental health [12].

The World Health Organization [13] and the European Union [14] have warned of the importance of promoting MHL in children and adolescents [9] through comprehensive programs based on scientific evidence, since this life stage seems to favor the occurrence of changes in their beliefs and behaviors [15], with encouraging results [16, 17] in terms of increasing knowledge about mental health. However, there is still a significant low to moderate levels of MHL [10, 15] thus the implementation of interventions that contribute to a direct and positive impact on adult life continues to be a necessity [18, 19].

Due to their characteristics, schools become an ideal place to reach a large number of children and adolescents [20], opening a safe space for a wide range of interventions [21], focused on the promotion and prevention of mental health and reduction of stigma in this population [9, 21, 22]. Research has increasingly shown [23–26] that interventions applied in the school context have significantly improved MHL, facilitating monitoring and help-seeking, an increase in self-recognition of mental disorders and an improvement in the reduction of stigma associated with mental health problems [25]. These programs are, usually, interactive, include group dynamics, modules and videos adapted to the target group. In addition, they include the intervention of several professionals, from teachers to specialized health professionals [25].

Most programs in school settings are face-to-face [15, 27, 28] and have an effective impact on student knowledge for the identification of specific mental disorders and changes in behavior. Nevertheless, other studies also suggest that programs that use online activities/classes [29–31] can also contribute to the prevention and early intervention in different dimensions of mental health, especially in ADHD, autism spectrum disorder, anxiety, depression, post-traumatic stress disorder, psychosis and eating disorders in adolescents [32–34]. Online interventions have greater ease of access and a lower cost, making them an excellent resource for promoting MHL. Since youth has more ease and increasingly early access to the technological world, this online format appears as a good alternative to the way of delivering intervention programs [35]. Research in this area points to promising results in programs that combine face-to-face and online sessions [30].

In Portugal, there is a growing concern with the promotion of mental health and prevention of mental illness, according to the National Mental Health Plan [36]. Some initiatives have been implemented but there is still a lot of work to be done in this area [11, 37]. In this sense, the project “Bicho de 7 Cabeças” [38] was created by the Psychosocial Rehabilitation Laboratory from Rehabilitation Research Center, whose objective was to increase levels of MHL and reduce stigma regarding mental illness. In the first place, MHL and stigma levels were assessed in several target groups in order to develop specific interventions according to their concrete needs [39]. Consequently, the present study aims to measure the impact of a mental health literacy intervention program in b-learning format on the increase of knowledge and the decrease of stigma in young people from Póvoa de Varzim, a municipality in the north of Portugal.

## Methods

### Study design

A quasi-experimental study was conducted involving one experimental group and an active control group, utilizing a pre-test/post-test design [40, 41] between November 2022 and May 2023.

### Participants

**Fig. 1:**
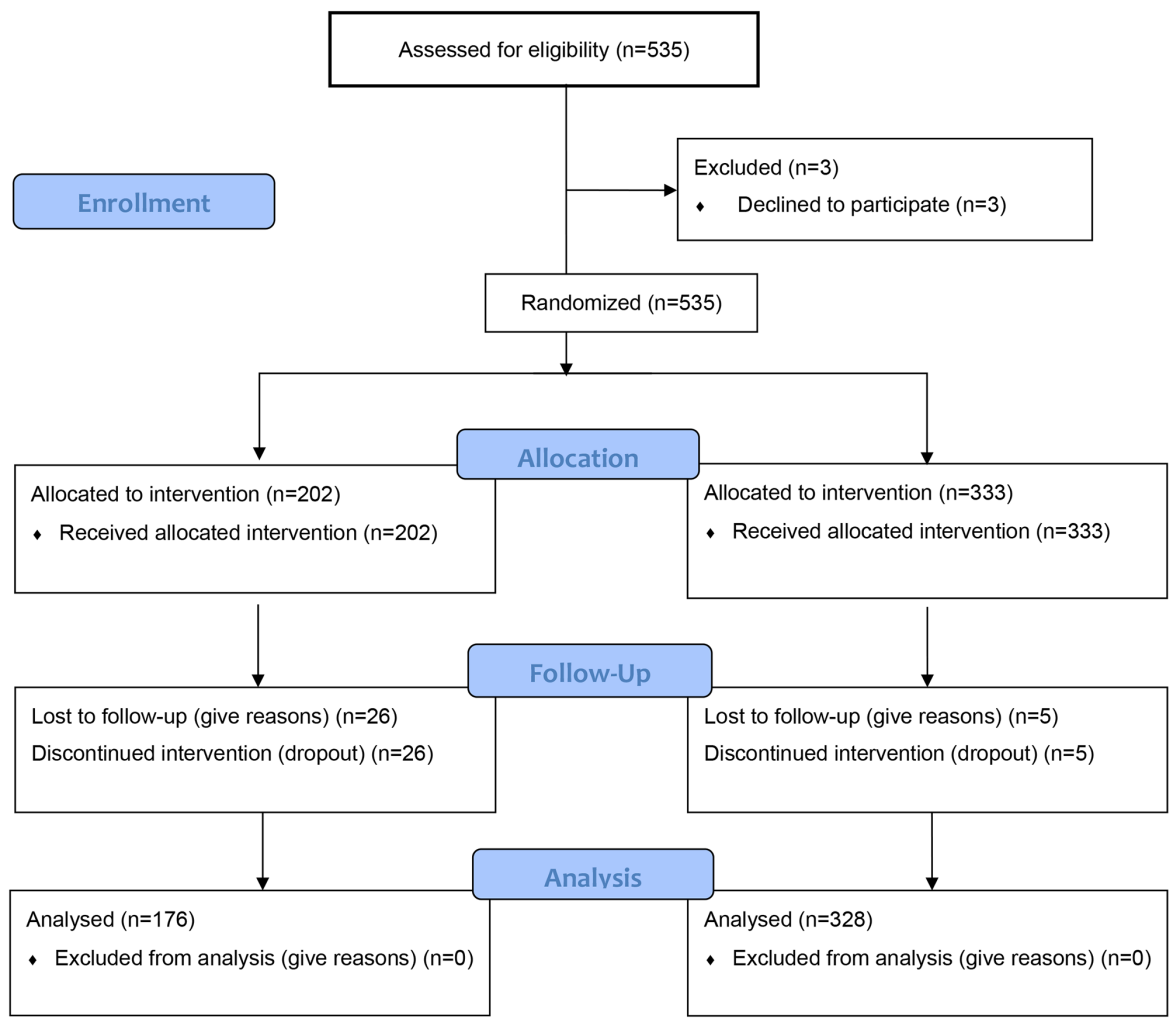
Flowchart of the allocation of participants

Participants were selected through a non-probabilistic convenience sampling method [42], drawn from the population of youth attending six public schools in Póvoa de Varzim. Out of these schools that agreed to participate in the study, the classes were randomly assigned to the control and experimental groups using a simple randomisation method that involved drawing lots for each class to be allocated to one group or the other [43]. Young students from the 9th grade were defined as inclusion criteria, since they were in the transition from latter stages of early adolescence to middle adolescence, where they started to show higher levels of autonomy and self-control and also the establishement of more stable emotional and social bounds [15, 44, 45]. Exclusion criteria included having communication and/or cognitive deficits that would hinder the comprehension of the questions. The recruitment and sample selection process was described using the CONSORT diagram [46], illustrating the progress of all participants through the different phases of the study (Fig. 1).

### Instruments

As a data collection method, an online self-administered questionnaire was employed, using the “Microsoft Forms” platform. The questionnaire encompassed the following information: (a) sociodemographic details, including age and gender; (b) MHPK-10; (c) MHLM; (d) MAKS, (e) RIBS and (f) CAMI. Additionally, the questionnaire included an informed consent form directed towards the parents/ guardians of the participants.

The Portuguese version of the Mental Health Promoting Knowledge Scale - MHPK-10 [47, 48] was used. This instrument aims to assess individuals’ knowledge regarding factors that promote good mental health. It comprises 10 statements that need to be rated based on their truthfulness, according to the individual’s perception of their importance in maintaining good mental health. Each question offers six response options, ranging from “Completely wrong” − 1 to “Completely correct” − 5, along with the “Don’t know” option, scored as zero. The final score of the instrument is calculated by averaging the scores of all ten questions [47]. In its original version, this scale demonstrates a good internal consistency (Cronbach’s α = 0.84), with the Portuguese version having a slightly lower value (Cronbach’s α = 0.79) [48].

The Portuguese version of the Mental Health Literacy Measure (MHLM) [49, 50] was employed. This instrument aims to assess levels of Mental Health Literacy (MHL). It consists of 26 items, divided into three components: Knowledge (12 items), Beliefs (22 items), and Resources (26 items). The first 22 questions are rated on a 5-point Likert scale, ranging from “Strongly disagree” − 1 to “Strongly agree” − 5. For the first 12 questions, 1 point is assigned to the last two options and 0 to the others. For the following 10 questions, 1 point is given for the last two options and 0 for the rest. The last four questions are assessed on a dichotomous scale of “yes” − 1 and “no” − 0. The total score ranges from 0 to 26 points, with a higher score indicating a higher level of literacy. In its original version [49], this scale demonstrates good internal consistency (Cronbach’s α = 0.83), and the Portuguese version has an equivalent value (Cronbach’s α = 0.81) [50].

The Portuguese version of the Mental Illness Knowledge Schedule (MAKS) [51, 52] was used. This instrument aims to assess knowledge related to mental health. It comprises 12 items divided into two parts. The first part, consisting of the initial six items, assesses knowledge regarding various factors associated with stigma in mental illness. The second part consists of the remaining six items, aimed at evaluating knowledge about specific mental illnesses. All items are rated using a 5-point Likert scale. A higher total score indicates a higher level of knowledge. In its original version [52], this scale demonstrates weak internal consistency (Cronbach’s α = 0.65), and the validation for the Portuguese version yielded an even lower internal consistency than the original (Cronbach’s α = 0.285) [51].

The Portuguese version of the Reported and Intended Behaviour Scale (RIBS) [53, 54] was employed. This instrument aims to assess the experiences and viewpoints of the population regarding individuals with mental illness. It consists of 8 items, with the first four exploring the experience of interacting with people with mental illness, and the latter four evaluating future perspectives in the aforementioned domains. The initial four questions, designed to understand the experience with people with mental illness, are answered using a dichotomous scale - “Yes” and “No”. The final items are assessed using a 5-point Likert scale, ranging from 1 ="Strongly disagree” to 5 ="Strongly agree”. A higher score indicates a lower stigma towards people with mental illness. RIBS has a good reliability (test-retest reliability = 0.75 and an Cronbach’s alpha = 0.85) [53]. The Portuguese version of the RIBS obtained a slighlty lower internal consistency than the original (Cronbach’s α = 0.81) [54].

The Portuguese version of the Community Attitudes toward People with Mental Illness (CAMI) [55, 56] consists of 27 items and is categorized into 3 subscales: attitudes about social exclusion, feelings of benevolence, tolerance, and support for care in community mental health. It includes 26 statements and an additional item concerning attitudes towards employment. Respondents provide answers on a 5-point Likert scale (ranging from 1= “Strongly Agree” to 5 = “Strongly Disagree”), and these responses are summed to yield a total score. A higher total score on the CAMI indicates fewer stigmatizing attitudes within the community. The Cronbach’s α values for the two components of the Portuguese version are: “Prejudices and Exclusion” α = 0.70, “Tolerance and Support in the Community” α = 0.63 [55].

### Ethical considerations

The study was approved by the ESS, Polytechnic University of Porto Ethics Committee (Nº 1748). It is a part of a broader research conducted by Psychosocial Rehabilitation Laboratory from Rehabilitation Research Center in collaboration with the Municipality of Póvoa de Varzim, aimed at enhancing mental health literacy within the local population. All participants or their legal guardians, in the case of minors, formally consented to participate in the study through an informed consent form, in accordance with the principles outlined in the “Declaration of Helsinki” [57]. This document detailed the study objectives, ensured voluntary participation, safeguarded participant privacy and data confidentiality, and conveyed the option to withdraw from the study without facing any penalties.

### Procedures

An online questionnaire with the Mental Health Promoting Knowledge (MHPK-10), Mental Health Literacy Measure (MHLM), Mental Illness Knowledge Schedule (MAKS), Community Attitudes toward People with Mental Illness (CAMI), and Reported and Intended Behaviour Scale (RIBS) was created using Microsoft Forms. Data collection took place at schools, during a Citizenship class, with the support of a member from the research team. The average time spent in survey completion was 15 min.

The division of the youth into experimental and control groups was achieved through a random draw of 9th-grade classes from the Schools of Póvoa de Varzim. The instruments (MHPK-10, MHLM, MAKS, CAMI and RIBS) were administered to the participants in the first and last sessions.

The Mental Health Literacy program consisted of the following components:

I. Experimental Group (EG)– Blended learning instructional sessions: Four face-to-face sessions and two asynchronous online sessions were conducted using the Zoom platform, each lasting between 40 and 45 min. The complete program comprised a total of six sessions. The first and last sessions were dedicated to assessment, while the third and fourth sessions were conducted online, with the remaining sessions being face-to-face. The first session took place one week before the intervention and the complete program lasted 6 weeks. Throughout all sessions, students were guided by both teachers and a mental health professional: the asynchronous online sessions were supervised by the teachers, during their classes; the face-to-face sessions were conducted by a mental health professional. Both received training from the research team.

The intervention was strategically designed to begin with an introduction to the “Bicho de 7 Cabeças” project, followed by an initial assessment. Subsequent topics covered included “Mental Health vs. Mental Illness.” The second session held face-to-face focused on anxiety disorders. The third session, conducted asynchronously online, covered ADHD, while the fourth online session addressed autism spectrum disorder. The fifth session, held face-to-face, explored the topic of depression. Finally, the sixth face-to-face session encompassed the final evaluation and student reflection on the project. All sessions were interactive, incorporated group dynamics, and utilized audiovisual resources tailored to the target audience. It is important to note that the decision to include online sessions was influenced by the school and teachers’ availability, aiming to facilitate integration with the existing curriculum.

II. Control Group (CG)– An active control group was chosen, and the program for this group comprised two face-to-face sessions: an initial assessment session and a final assessment session for instrument administration. The remaining intervention was designed so that after the initial assessment, informational brochures about mental health and certain mental disorders were provided to each responsible teacher of the classes. The intention was for these brochures to be distributed to the participants.

The Supplementary Material 1 outlines the organization of the programs for each group.

### Statistical analysis

The data from the online questionnaires were automatically exported to Microsoft Office Excel, where the procedures for coding the variables were performed. Next, for data processing, the data were exported to the IBM Statistical Package for the Social Sciences (SPSS) 28.0, considering a significance level of 0.05 for all statistical tests performed [42, 58]. Descriptive statistics were used to characterize the sample using measures of central tendency and dispersion: mean (x) and standard deviation (SD) for continuous or discrete variables and relative frequencies (%) for nominal or ordinal data. As the sample has *n* > 300, it was necessary to test normality using the absolute values of asymmetry and kurtosis, since the Kolmogorov-Smirnov normality test may not be feasible for samples of this size [58]. For inferential statistics, parametric tests were used whenever data distribution was deemed approximately normal using threshold criteria for skewness and kurtosis - less than|2.0| and|9.0|, respectively [59]. Additionally, one-way repeated-measures ANOVA were implemented for comparing the pre-post conditions. For these models, sphericity was tested using Mauchly’s test. The Huynh-Feldt correction was employed whenever this assumption was not met and the epsilon was higher than 0.57; otherwise, the Greenhouse-Geisser correction was used [59].

## Results

Table 1 shows the characteristics of the participants. The control group (*n* = 176) had a slightly lower mean age (14.15 ± 0.65) than the experimental group (14.27 ± 0.66) and, although this difference is statistically significant (*p* =.049) the small difference may be overlooked. The experimental group (*n* = 328) has a higher percentage (57.30%) of female participants than the control group (52.80%) however, this difference is not statistically significant (*p* =.348).

**Table 1:** Characterization of participants

Variables		Total *n* = 504	Experimental *n* = 328	Control *n* = 176		95% CI
		$\bar {x}$ ± SD	$\bar {x}$ ± SD	$\bar {x}$ ± SD	*p*-value	
**Age (years)**		14.23 ± 0.66	14.27 ± 0.66	14.15 ± 0.65	0.049*	[0.001; 0.240]
		**n %**	**n (%)**	**n (%)**	***p-value***	
**Gender**	Female	281 (55.80) 223 (44.20)	188 (57.30)	93 (52.80) 83 (47.20)	0.348*	
	Male		140 (42.70)			

$\bar {x}$– mean; SD– standard deviation; n = absolute frequency; % = relative frequency; *Independent samples T-test **Fisher’s exact test; CI– Confidance interval

Table 2 shows the simple comparisons between and within groups. It can be seen that there are differences at baseline between the two groups in the RIBS variables (where the control group had less stigma attitudes − 8.60 ± 3.31 vs. 7.48 ± 2.96 in the experimental; *p* =.006; CI [-1.42; -0.24]) and in the MHLM (the experimental group had higher mental health literacy − 32.00 ± 10.32 than the control − 28.80 ± 11.78; *p* =.003; CI [1.13; 5.28]). After the intervention, there were differences between the two groups in the RIBS variables (with the experimental group now showing fewer stigma attitudes − 16.55 ± 3.14 vs. 15.67 ± 3.26 in the control; *p* =.003; CI [0.29; 1.46]) and in the MAKS (the experimental group showed higher mental health literacy − 42.16 ± 8.38 than the control − 39.24 ± 8.24; *p* <.001; CI [1.39; 4.45]).

**Table 2:** Values of mental health literacy and stigma in both groups

Variables		Experimental	Control		
		$\bar {x}$ ± SD	$\bar {x}$ ± SD	*p*-value*	95% CI
RIBS	Pre-test	7.78 ± 3.13	8.61 ± 3.31	0.006	[-1.42; -0.24]
	Post-test	16.55 ± 3.14	15.67 ± 3.26	0.003	[0.29; 1.46]
		< 0.001	< 0.001		
CAMI	Pre-test	88.22 ± 9.25	80.49 ± 10.18	0.054	[-0.03; 3.48]
	Post-test	88.91 ± 11.58	83.25 ± 9.39	0.737	[-2.34; 1.65]
		0.020	0.349		
MAKS	Pre-test	39.41 ± 9.53	38.06 ± 10.34	0.141	[-0.45; 3.15]
	Post-test	42.16 ± 8.38	39.24 ± 8.24	< 0.001	[1.39; 4.45]
		< 0.001	< 0.001		
MHPK	Pre-test	15.39 ± 6.32	15.51 ± 4.95	0.830	[-1.20; 0.26]
	Post-test	15.53 ± 8.30	16.11 ± 7.29	0.440	[-2.04; 0.89]
		0.479	0.787		
MHLM	Pre-test	32.00 ± 10.32	28.80 ± 11.78	0.003	[1.13; 5.28]
	Post-test	31.86 ± 11.48	31.96 ± 9.59	0.916	[-1.98; 1.78]
		0.092	0.843		

$\bar {x}$ - mean; sd– standard deviation; *Independent samples T-test; MHPK-10 - Mental Health Promoting Knowledge; MHLM - Mental Health Literacy Measure; MAKS - Mental Illness Knowledge Schedule; RIBS - Reported and Intended Behaviour Scale; CI– confidence interval

Within the experimental group, there were improvements from the first to the second assessment in the variables RIBS (from 7.78 ± 3.13 to 16.55 ± 3.14; *p* <.001), CAMI (from 88.22 ± 9.25 to 88.91 ± 11.58; *p* =.020) and MAKS (from 39.41 ± 9.53 to 42.16 ± 8.38; *p* <.001). In the control group there were also differences between the first and second assessments, namely in RIBS (from 8.61 ± 3.31 to 15.67 ± 3.26; *p* <.001) and MAKS (from 38.06 ± 10.34 to 39.24 ± 8.24; *p* <.001).

Table 3 shows that there is a significant difference between the stigma scores (RIBS) at baseline (*p* <.001, with a medium effect size - η2 = 0.679) and follow-up (*p* <.001, with a small effect size - η2 = 0.024), and the interaction between the intervention and the time elapsed between the two assessments is also significant (*p* <.001). Regarding knowledge (MAKS), there are also significant differences between the scores at baseline (*p* <.001, with a small effect size - η2 = 0.029), but the interaction between the intervention effect and time is not statistically significant (*p* =.126, with a small effect size - η2 = 0.005). The remaining assessment instruments did not show significant values.

**Table 3:** Effects of intervention in mental health literacy and stigma

Variables		Experimental	Control						
		$\bar {\bf x}$ ± **SD**	$\bar {\bf x}$ ± **SD**	***p-value*** ^a^	***Observed power*** *	***η***2***#***	***p-value*** ^b^	Observed power**	***η***2***##***
RIBS	Pre-test	7.78 ± 3.13	8.61 ± 3.31	< 0.001	1.000	0.679	< 0.001	0.939	0.024
	Post-test	16.55 ± 3.14	15.67 ± 3.26						
MAKS	Pre-test	39.41 ± 9.53	38.06 ± 10.34	< 0.001	0.970	0.029	0.126	0.333	0.005
	Post-test	42.16 ± 8.38	39.24 ± 8.24						

$\bar {\bf x}$ - mean; SD– standard deviation; ^a^ within-groups *p*-value; ^b^ interaction p-value; * within-groups observed power; ** interaction oberserved power; MAKS - Mental Illness Knowledge Schedule; RIBS - Reported and Intended Behaviour Scale; # η2 within-groups partial eta squared; ## η2 interaction partial eta squared

## Discussion

The present study aimed to measure the impact of a mental health literacy intervention program on the increase of knowledge and the decrease of stigma in young people from Póvoa de Varzim. For this, 504 9th grade students from Schools of this municipality participated, and an increase in MHL and a decrease in stigma in both groups was observed, more significant in the experimental group.

The results of our program are in agreement with several studies [60, 61] that prove that interventions aimed at children/adolescents for the promotion of mental health are effective in promoting empowerment, contributing to the increase of knowledge in MHL, decreasing stigma, seeking help, early detection of mental disorders, in order to mitigate the lifelong consequences that prevent them from leading a full life [62]. Several studies [11, 63] also highlight the importance of implementing programs to increase MHL and decrease stigma in school environments as early as possible, in order to foster healthy lifestyles in the population from childhood and adolescence.

In terms of the increase in MHL knowledge, our findings indicated that both groups improved, which is positive for the intervention as a whole. However, when comparing the groups, it is clear that the experimental group obtained greater MHL than the control group, not only due to the effect of time, but in its interaction with the intervention carried out. These results are in line with those of similar studies, where a program to increase MHL has been shown to have a positive impact on students who received the intervention, resulting in a greater increase in MHL, help-seeking and stigma reduction [64, 65].

Concerning the influence of the increase in MHL on the reduction of stigma, the results of this study show that both groups improved, which can be explained by both groups having access to an intervention, the control group having access to information leaflets and the experimental group having access to educational sessions [66]. Although, it is clear that in the experimental group, the decrease in stigma from pre to post-intervention was significantly greater than in the control group, proving the consensus that programs that aim to improve the knowledge, attitudes and and beliefs regarding mental disorders can lead to the reduction of stigma [16, 26, 60, 61]. Additionally, regardless of whether it was a control or experimental group, both interventions used clear, youthful language and attractive design, which may have motivated young people both to attend the sessions and to explore the informative brochures. Moreover, and according to a recent study, adolescent mental health literacy can be improved by the provision of the necessary resources and facilities, ongoing evaluation of interventions, and the dissemination of information about the services offered [67], actions the authors have taken in this project.

By strategically addressing both knowledge enhancement and stigma reduction, individuals gain a more comprehensive understanding of mental health, since increasing literacy ensures accurate knowledge about mental disorders, while stigma reduction fosters empathy and understanding, contributing to a well-rounded perspective [17]. Moreover, combining literacy promotion and stigma reduction strategies can lead to more effective and long-term behavioral changes, because people who are well-informed about mental health issues and harbor fewer stigmatizing beliefs are more likely to engage with and support those experiencing mental health challenges which contributes to building a more inclusive and compassionate community.

The present study focused on the intervention with 9th grade students. The results showed that, in fact, the intervention in this age group produced significant changes after the intervention. According to Skre, Friborg [68], younger adolescents have less knowledge about the symptoms of mental disorders, which may indicate that they are not cognitively prepared to understand mental disorders. In addition to low literacy, younger participants, as expected, exhibit more prejudice and stereotypes, possibly due to their identity not being sufficiently mature [68]. It is believed that the fact that education is the most effective method in young people can be explained by the greater development of their cognitive flexibility compared to adults [69]. Cognitive flexibility allows young people to have a more open mind and less prone to rigid and immutable ideas [69]. It is important to also consider that, school-based mental health interventions are valued and helpful, although in a qualitative study some students have expressed worries about feeling exposed or vulnerable in the classroom [70]. Nevertheless, some authors argue that there should be a movement toward integrating mental health systems within the education sector, including training the teaching staff to identify signs of mental health problems in their students and stigma situations, integrating school and community mental health systems to develop a full continuum of mental health supports for young people [71].

The present study has some limitations, such as the fact that the program is short-term and does not have a follow-up post-intervention, which makes it insufficient to assess whether the positive impact of the interventions has been maintained over time, in order to modify behaviors in a lasting way. Thus, although there is a pre-test and post-test evaluation, these evaluations were carried out shortly after the intervention, which can lead to generalizable results, and not positive and lasting results [26, 72]. As a suggestion, a follow-up each year till the end of the high school is recommended. Still, despite the promising results, not all the instruments used revealed significant results in relation to knowledge in MHL, namely the MHLM, CAMI and MHPK-10 instruments. Such results may come from the extension of the instruments applied and, since these three instruments were the last to be answered, it may have led to the participants feeling tired and not paying due attention to what they were answering. This type of situation is common and influences the results obtained [73]. It is also important to acknowledge a potential source of bias, namely the lack of information about own experience of mental illness or contact with people with mental illness (information that the authors did no collect due to the nature of this setting and to preserve students’ privacy).

Although, in this study, some significant improvements are found in the increase in knowledge in MHL and its relationship with the reduction of stigma, the magnitude of these changes was small, when compared to those found by Milin and collaborators [74]. It is still not clear how these small MHL-related effects can translate into changes in actual mental health levels, both at the individual and group levels. Additionaly, it is suggested that there could be a refresher intervention for these students, for example, in high school, before entering higher education or entering active life (the job market). In order to properly implement policies that can change profoundly young people’s knowledge and perceptions regarding mental health and mental illness, policymakers should pay close attention to innitiatives like “Bicho de 7 Cabeças” [38], as these can effectively make communities healthier and more aware to these issues.

## Conclusion

This study measured the impact of a b-learning school-based program, called “Bicho de 7 Cabeças”, which aimed to increase mental health knowledge and decrease stigma towards mental illnesses in young people from Póvoa de Varzim, a municipality in the north of Portugal.

The comparison of the results between the experimental group and the control group showed that the intervention program allowed participants to acquire a greater knowledge about mental health and reduce stigma related to mental disorders. The results of the present study suggest that a school-based b-learning program can be effective in increasing mental health literacy and reducing stigma in young people. Those results are in agreement with several studies that proves that this type of intervention, to promote mental health literacy aimed at children/adolescents increases knowledge and decreases stigma towards people with mental illness. As a result, the policies and campaigns being established by the municipality of Póvoa de Varzim should continue and should be seen as a good practice to be implemented elsewhere, always respecting the particular characteristics of each group, in order to adapt the strategies to be used.

### Relevance for clinical practice

This study demonstrates the tangible benefits of mental health literacy programs, specifically the “Bicho de 7 Cabeças” project in a b-learning format, for young people. Nurses and other mental health professionals should considered these findings when designing programs to reduce stigma towards mental illness and promoting literacy. Since schools are the contexts in which this population spends most of its time, they are excellent places to implement these initiatives and ensure that possible mental health problems can be prevented and detected early.

### Electronic supplementary material

Below is the link to the electronic supplementary material.

Supplementary Material 1

## Data Availability

The data that support the findings of this study are available from the one of the authors, MJT, upon reasonable request.
