# Supplementary material for: Assessing the efficacy of the ‘Bicho De 7 Cabeças’ B-learning school-based program in enhancing mental health literacy and reducing stigma
Source: BMC Psychol. 2024 Feb 23;12:93. doi: 10.1186/s40359-024-01591-2 (PMC10893733; doi:10.1186/s40359-024-01591-2)
Supplement: Supplementary file 1 — Supplementary Material 1 [file 40359_2024_1591_MOESM1_ESM.docx]

Supplementary Material 1

Intervention Applied to Experimental Group (GE) and Control Group (GC)

|  | **GROUP 1 – Experimental**  **4 Face-to-face Sessions and 2 Asynchronous Online Sessions** | **GROUP 2 – Control**  **2 Face-to-face Sessions with Brochures** |
| --- | --- | --- |
| Session 1  Face-to-face | Presentation of the "Bicho 7 Cabeças" project + Initial Assessment; Exploration of the topic Mental Health vs. Mental Illness | Initial Assessment + Brochure handed out by teachers to students; Mental Health Brochure: Living with mental illness, warning signs, causes and risk factors, intervention, seeking help, and coping with the condition. |
| Session 2 Synchronous Online | Attention-Deficit/Hyperactivity Disorder (ADHD): What it is; warning signs and seeking help; the causes; intervention and how to cope and provide support. | ADHD Brochure: Understanding the condition, warning signs, causes and risk factors, intervention, seeking help, and coping with the condition. |
| Session 3  Face-to-face | Anxiety Disorder (AD): What it is; warning signs and seeking help; the causes; intervention and how to cope and provide support. Suggested strategy (diaphragmatic breathing). | AD Brochure: Understanding the condition, warning signs, causes and risk factors, intervention, seeking help, and coping with the condition. |
| Session 4  Synchronous Online | Autism Spectrum Disorder (ASD): What it is; warning signs and seeking help; the causes; intervention and how to cope and provide support. | ASD Brochure: Understanding the condition, warning signs, causes and risk factors, intervention, seeking help, and coping with the condition. |
| Session 5  Face-to-face | Depression: What it is; warning signs and seeking help; the causes; intervention and how to cope and provide support. | Depression Brochure: Understanding the condition, warning signs, causes and risk factors, intervention, seeking help, and coping with the condition. |
| Session 6  Face-to-face | Final Assessment + Project Reflection | Final assessment |
| Note: Both groups had access to the project website: https://www.labrp.pt/setecabecas2/ | | |
